# Hyperparameter Optimization EM Algorithm via Bayesian Optimization and Relative Entropy

**DOI:** 10.3390/e27070678

**Published:** 2025-06-25

**Authors:** Dawei Zou, Chunhua Ma, Peng Wang, Yanqiu Geng

**Affiliations:** 1 School of Information Engineering, Suihua University, Suihua 152061, China; zoudawei0928@gmail.com (D.Z.);; 2 Engineering Technology Research Center of Artificial Intelligence Innovation Application, Suihua University, Suihua 152061, China

**Keywords:** hyperparameters optimization, evidence function, relative entropy, EM algorithm

## Abstract

Hyperparameter optimization (HPO), which is also called hyperparameter tuning, is a vital component of developing machine learning models. These parameters, which regulate the behavior of the machine learning algorithm and cannot be directly learned from the given training data, can significantly affect the performance of the model. In the context of relevance vector machine hyperparameter optimization, we have used zero-mean Gaussian weight priors to derive iterative equations through evidence function maximization. For a general Gaussian weight prior and Bayesian linear regression, we similarly derive iterative reestimation equations for hyperparameters through evidence function maximization. Subsequently, after using relative entropy and Bayesian optimization, the aforementioned non-closed-form reestimation equations can be partitioned into E and M steps, providing a clear mathematical and statistical explanation for the iterative reestimation equations of hyperparameters. The experimental result shows the effectiveness of the EM algorithm of hyperparameter optimization, and the algorithm also has the merit of fast convergence, except that the covariance of the posterior distribution is a singular matrix, which affects the increase in the likelihood.

## 1. Introduction

In machine learning, parameters are classified into two categories: model parameters, which are internal and configurable, and hyperparameters, which are external and cannot be estimated from data. Model parameters include the weights in a deep neural network, for example. Hyperparameters include batch size, the learning rate, and the number of hidden layers in a neural network [1]. Hyperparameter optimization (HPO) represents a pivotal aspect of machine learning model training, including the process of fine-tuning the hyperparameters of a model to improve its performance. Hyperparameters are parameters that are set before the learning process begins. The process of tuning these hyperparameters can significantly affect the model’s accuracy and generalization ability [2]. There are some main hyperparameter optimization methods as follows. Grid search: Grid search is the brute-force way of searching hyperparameters [3], with defined lower and higher bounds along with specific steps [4]. Grid search works based on the Cartesian product of the different sets of values, evaluates every configuration, and returns the combination with the best performance [5]. Grid search is a simple implementation that, however, can be highly inefficient for large search spaces due to its exhaustive nature. This problem is further compounded as data dimensionality increases. Random search: Random search involves randomly sampling hyperparameter combinations from a predefined search space. While less computationally intensive than grid search, it can often identify superior hyperparameter configurations because of its ability to explore the search space more efficiently [6,7]. Genetic algorithms: Genetic algorithms are inspired by the process of natural selection, whereby a population of candidate solutions (hyperparameter configurations) evolves over multiple generations [8,9]. They are useful for exploring large search spaces and can handle discrete and nonlinear search spaces effectively. Gradient-based optimization: Gradient-based optimization treats hyperparameters as continuous variables [10,11,12]. This approach is often used in neural network architectures and can be efficient for optimizing large-scale models. Bayesian optimization: Bayesian optimization employs a probabilistic model of the objective function (model performance) to identify the most promising hyperparameter configurations for evaluation. This approach is more efficient than grid and random search, particularly in the context of high-dimensional and noisy optimization problems [13,14,15,16]. Recently, automated machine learning (AutoML) platforms also have been applied to practical problems, which automate the entire machine learning pipeline, including data preprocessing, model selection, feature engineering, and hyperparameter optimization. They leverage various optimization techniques to find the best model configuration automatically and make machine learning more accessible to users with limited expertise in data science and machine learning by automating repetitive tasks and reducing the need for manual intervention. This allows experts to focus more on understanding the problem domain and interpreting the results rather than spending time on technical details [17,18,19,20]. Particle swarm optimization (PSO) is a popular algorithm for hyperparameter optimization recently. It is simple to implement and explores the global search space efficiently [21,22,23]. However, methods based on gradients, grid search, random search, genetic algorithms, and particle swarm optimization all lack rigorous mathematical explanation; they are more computational logic. Additionally, grid search, random search, and particle swarm optimization are all brute-force methods that are time-consuming and labor-intensive. Our proposed hyperparameter optimization EM algorithm, based on Bayesian optimization theory and relative entropy, has a strict mathematical derivation and explanation. The simulation results show that the algorithm has the advantage of fast convergence.

The relevance vector machine (RVM) represents a Bayesian sparse kernel technique that has been developed for the purpose of regression tasks. The RVM model for regression is linear, whose weight prior is Gaussian, defined by the following form [24]

(1) $$p(\omega |\eta )=\prod_{i=1}^{M}N(\omega_{i}|0,\eta_{i}^{-1}),$$

where $\eta_{i}$ is a hyperparameter and $\eta ={\left(\eta_{1},\ldots ,\eta_{M}\right)}^{T}$.

In this paper, we will propose a Bayesian hyperparameter optimization in terms of a more general form of weight prior, defined as follows:

(2) $$p(\omega |\eta ,\mu )=\prod_{i=1}^{M}N(\omega_{i}|\mu_{i},\eta_{i}^{-1}),$$

where $\mu_{i}$ is also a hyperparameter and $\mu ={\left(\mu_{1},\ldots ,\mu_{M}\right)}^{T}$.

We designate weight prior (2) as the general Gaussian weight prior (GGWP). Initially, leveraging evidence function maximization and GGWP, we derive non-closed-form iteratively reestimation equations for hyperparameters. Subsequently, we partition the aforementioned non-closed-form reestimation equations into E step and M step, elucidating the hyperparameter reestimation equations mathematically and statistically.

## 2. Related Mathematical Knowledge

**Lemma** **1.**  $\frac{\partial }{\partial x}\ln\left|M\right|=Tr(M^{-1}\frac{\partial M}{\partial x})$.

**Lemma** **2.**  $\frac{\partial }{\partial x}(M^{-1})=-M^{-1}\frac{\partial M}{\partial x}M^{-1}$.

The proofs of both Lemmas 1 and 2 refer to [24,25,26,27]

**Definition** **1.** *Let *∇* be gradient and *$h^{T}\nabla$* is the operator such that for *k∈N*,*

$${\left(h^{T}\nabla \right)}^{k+1}f=(h^{T}\nabla ){\left(h^{T}\nabla \right)}^{k}f,$$

*where *${\left(h^{T}\nabla \right)}^{0}f=f$.

The symbolic powers are referred to as operators that act upon a multivariate function with n variables.

**Lemma** **3** (multivariate Taylor’s expansion)**.** *Let *$f:B\left(x,r\right)\to \mathbb{R}$* be a multivariate function, where* $B\left(x,r\right)\subseteq {\mathbb{R}}^{k}$* and* $f(x)\in C\left(B\left(x,r\right)\right)$*. For* $h\in {\mathbb{R}}^{k}$ $\left(∥h∥<r\right)$*, then there exists* θ∈(0,1)* such that *

$$f\left(x+h\right)=\overset{n-1}{\underset{l=0}{\sum }}\frac{1}{l!}({\left(h^{T}\nabla \right)}^{l}f)(x)+\frac{1}{n!}({\left(h^{T}\nabla \right)}^{n}f)(x+\theta h).$$

Both Definition 1 and proof of Lemma 3 in detail refer to [28].

**Corollary** **1.** *A quadratic function* $f:B\left(w,r\right)\to \mathbb{R}$ *is defined as follows:*

$$f(x)=x^{T}Ax+x^{T}b+c,$$

*where* c *is a scalar constant,* b *and* $x={\left(x_{1},\ldots ,x_{n}\right)}^{T}$ *are both n-dimensional column vectors as well as* A *is a n-dimensional inverse and symmetric matrix. Then, at the stationary point* $x_{0}=\frac{1}{2}A^{-1}b$ *, we obtain the Taylor expansion of the function* f(x) *as follows:*

$$f(x)=f(x_{0})+\frac{1}{2}{\left(x-x_{0}\right)}^{T}H(x-x_{0}),$$

*where elements of the Hessian matrix* H *are defined by*

$$\left(\frac{\partial f(x)}{\partial x_{i}\partial x_{j}}\right).$$

The proof of Corollary 1 refers to [29] in detail.

## 3. HPO via Maximization of the Evidence Function

### 3.1. Bayesian Linear Regression and Linear Basis Function Models

The linear model for regression is given with linear combinations of fixed nonlinear functions of the input variables as follows:

(3) $$y(x,\omega )=\omega_{0}+\sum_{i=1}^{M-1}\omega_{i}\psi_{i}(x),$$

where $\omega_{0}$ is called bias parameter and $\psi_{i}(x)$ is a basis function. For the sake of convenience, setting $\psi_{0}(x)=1$.

We rewrite (3) in matrix form [24]:

(4) $$y(x,\omega )=\omega^{T}\psi (x),$$

where $\psi (x)={\left(\psi_{0}(x),\ldots ,\psi_{M-1}(x)\right)}^{T}$ and $\omega ={\left(\omega_{0},\ldots ,\omega_{M-1}\right)}^{T}$.

The target variable t for regression is defined as the sum of a random noise ε and a deterministic function y(x,ω) as follows:

(5) t=y(x,ω)+ε,

where the noise ε is Gaussian, whose mean is zero and precision is λ (inverse variance). So, we obtain

(6) $$p(t|x,\omega ,\lambda )=N(t|y(x,\omega ),\lambda^{-1}).$$

Now, $X=(x_{1},\ldots ,x_{N})$, called the input dataset, is now considered in conjunction with its target value vector $T=(t_{1},\ldots ,t_{N})$. These data points are all drawn independently from the Gaussian distribution (6) based on our assumption. Thus, the likelihood function is obtained as follows:

(7) $$p(T|X,\omega ,\lambda )=\prod_{i=1}^{N}N(t_{i}|\omega^{T}\psi (x_{i}),\lambda^{-1}).$$

According to (7), we define corresponding conjugate prior as follows:

(8) $$p(\omega )=N(\omega |m_{0},C_{0})$$

From (7) and (8), we can shortly obtain the following posterior distribution:

(9) $$p(\omega |T,X,\lambda )=N(\omega |m_{N},C_{N}),$$

where

(10) $$m_{N}=C_{N}(C_{0}^{-1}m_{0}+\lambda \Psi^{T}t),$$

(11) $$C_{N}^{-1}=C_{0}^{-1}+\lambda \Psi^{T}\Psi ,$$

(12) $$\Psi =\left(\begin{array}{ccc} \psi_{0}(x_{1}) & \ldots & \psi_{M-1}(x_{1})\\ \vdots & \ddots & \vdots \\ \psi_{0}(x_{N}) & \ldots & \psi_{M-1}(x_{N}) \end{array}\right).$$

The proofs of both (10) and (11) in detail refer to Appendix A in [29].

### 3.2. Evidence Approximation and Bayesian Model Comparison

Let us suppose we are presented with a series of models $\left\{M_{k}\right\}$, k=1,…,L. We compare these models and then choose the optimal one according to a Bayesian perspective, aiming to mitigate the risk of overfitting commonly associated with maximum likelihood approaches. We express uncertainty by a prior probability $p(M_{k})$. In the context of a training set, it is reasonable to assume that all models have the same prior probability. This assumption is consistent with the notion that, in practice, there should be no inherent preference for any specific models L. The objective is thus to assess the following posterior distribution given the dataset D,

(13) $$p(M_{k}|D)\propto p(M_{k})p(D|M_{k}).$$

The posterior distribution $p(M_{k}|D)$ represents the model evidence, also known as the marginal likelihood. This term is interpreted as a likelihood function [24].

In this section, we present a fully Bayesian treatment based on the introduction of priors over the hyperparameters η, λ and μ. The derivation of predictions can be accomplished by using the marginalization over these hyperparameters and the weight parameter. Nevertheless, it is difficult to completely marginalize all the variables in the analytical process. Consequently, we propose an approximation approach to identify these hyperparameters η, λ and μ by the maximization of the marginal likelihood function after integrating over these parameters. In statistics, the method is commonly referred to as empirical Bayes [30,31], type 2 maximum likelihood [32], or generalized likelihood [33]. In the field of machine learning, it is frequently referred to as evidence approximation. [24,34]. Furthermore, a hyperprior over the parameters η, λ and μ is introduced first. Subsequently, the following predictive distribution is obtained:

(14) p(t|T)=∫∫∫∫p(t|x,ω,λ)p(η,λ,μ|T)p(ω|T,η,λ,μ)dηdλdμdω,

where p(t|x,ω,λ) is given according to (6) and p(ω|T,η,λ,μ) is determined from (9).

If the posterior p(η,λ,μ|T) exhibits a sharp peak around $\hat{\eta }$, $\hat{\lambda }$ and $\hat{\mu }$, the predictive distribution can be derived by marginalizing over ω, where we set the hyperparameters to the value $\hat{\eta }$, $\hat{\lambda }$ and $\hat{\mu }$. So, we obtain

(15) $$p(t|T)≃p(t|T,\hat{\eta },\hat{\lambda },\hat{\mu })=\int p(\omega |T,\hat{\eta },\hat{\lambda },\hat{\mu })p(t|x,\omega ,\hat{\lambda })d\omega .$$

The posterior for the hyperparameters is expressed as

(16) p(η,λ,μ|T)∝p(η,λ,μ)p(T|η,λ,μ).

The values of $\hat{\eta }$, $\hat{\lambda }$, and $\hat{\mu }$ can be derived by the maximization of the marginal likelihood function p(T|η,λ,μ) based on the evidence approximation, particularly when the prior is relatively flat. By continuously evaluating the marginal likelihood, it is possible to identify its maxima, which enables the values of these hyperparameters to be determined solely from the training dataset.

### 3.3. The Evidence Function Evaluation

From (7), we derive the following result:

(17) $$\begin{array}{cc} p(T|X,\omega ,\lambda ) & =\prod_{i=1}^{N}N(t_{i}|\omega^{T}\psi (x_{i}),\lambda^{-1})\\ & =\sqrt{{\left(\frac{\lambda }{2\pi }\right)}^{N}}\exp(-\frac{\lambda }{2}\sum_{i=1}^{N}{\left(t_{i}-\omega^{T}\psi (x_{i})\right)}^{2})\\ & =\sqrt{{\left(\frac{\lambda }{2\pi }\right)}^{N}}\exp(-\frac{\lambda }{2}{\left\|T-\Psi \omega \right\|}^{2}). \end{array}$$

From (2), the following result is obtained:

(18) $$\begin{array}{cc} p(\omega |\eta ,\mu ) & =\prod_{i=1}^{M}N(\omega_{i}|\mu_{i},\eta_{i}^{-1})\\ & =\frac{\sqrt{\prod_{i=1}^{M}\eta_{i}}}{\sqrt{{\left(2\pi \right)}^{M}}}\exp(-\frac{1}{2}\sum_{i=1}^{M}\eta_{i}{\left(\omega_{i}-\mu_{i}\right)}^{2})\\ & =\frac{{\left|\Lambda \right|}^{\frac{1}{2}}}{\sqrt{{\left(2\pi \right)}^{M}}}\exp(-\frac{1}{2}{(\omega -\mu )}^{T}\Lambda (\omega -\mu )). \end{array}$$

It can be demonstrated that the posterior with respect to weight parameter ω is Gaussian as follows:

(19) p(ω|T,X,η,λ,μ)=N(ω|m,C),

where

(20) $$m={\left(\Lambda +\lambda \Psi^{T}\Psi \right)}^{-1}(\Lambda \mu +\lambda \Psi^{T}T),$$

(21) $$C={\left(\Lambda +\lambda \Psi^{T}\Psi \right)}^{-1},$$

where $\Lambda =diag(\eta_{i})$.

The proofs of both (20) and (21) are detailed in Appendix A.

The evidence approximation is utilized to evaluate the hyperparameters. We obtain the marginal likelihood function (evidence function) by integrating out the weight parameters as follows:

(22) p(T|X,η,λ,μ)=∫p(T| X,ω,λ)p(ω|η,μ)dω,

Then, we obtain

(23) $$p(T|X,\eta ,\lambda ,\mu )=\sqrt{{\left(\frac{\lambda }{2\pi }\right)}^{N}}{\left|\Lambda \right|}^{\frac{1}{2}}{\left|C\right|}^{\frac{1}{2}}\exp(-G(m)),$$

where $G(m)=\frac{\lambda }{2}{\left\|T-\Psi m\right\|}^{2}+\frac{1}{2}{\left(m-\mu \right)}^{T}\Lambda (m-\mu )$.

The proof of (23) is detailed in Appendix B.

In terms of (23), we take the logarithm of the marginal likelihood function, also called the log marginal likelihood function, and obtain the following result:

(24) $$\ln p(T|X,\eta ,\lambda ,\mu )=\frac{N}{2}\ln\lambda -G(m)+\frac{1}{2}\sum_{i=1}^{M}\ln\eta_{i}+\frac{1}{2}\ln\left|C\right|-\frac{N}{2}\ln(2\pi ).$$

For convenience, we abbreviate the marginal likelihood function as the likelihood and the log marginal likelihood function as the log likelihood. Subsequently, the log likelihood will be maximized.

### 3.4. The Evidence Function Maximization

Now, p(T|X,η,λ,μ) is maximized with respect to $\eta_{i}$. From (24) and Lemma 1, we derive the following result:

(25) $$\frac{\partial }{\partial \eta_{i}}\ln\left|C\right|=Tr(C^{-1}\frac{\partial C}{\partial \eta_{i}})=Tr((\Lambda +\lambda \Psi^{T}\Psi )\frac{\partial }{\partial \eta_{i}}({\left(\Lambda +\lambda \Psi^{T}\Psi \right)}^{-1})),$$

By utilization of (21), (25), and Lemma 1, we get the following result:

(26) $$\frac{\partial }{\partial \eta_{i}}\ln\left|C\right|=-Tr((\frac{\partial }{\partial \eta_{i}}(\Lambda +\lambda \Psi^{T}\Psi ))C),$$

where we have used

$$\frac{\partial }{\partial \eta_{i}}({\left(\Lambda +\lambda \Psi^{T}\Psi \right)}^{-1})=-{\left(\Lambda +\lambda \Psi^{T}\Psi \right)}^{-1}\frac{\partial }{\partial \eta_{i}}((\Lambda +\lambda \Psi^{T}\Psi )){\left(\Lambda +\lambda \Psi^{T}\Psi \right)}^{-1}.$$

By making use of (26), the following result was obtained

(27) $$\frac{\partial }{\partial \eta_{i}}\ln\left|C\right|=-C_{ii},$$

where $C_{ii}$ represents the *i*th component of the principal diagonal of the covariance matrix C.

The following result is obtained:

(28) $$\frac{\partial G(m)}{\partial \eta_{i}}=\frac{1}{2}m_{i}^{2},$$

By making use of (27) and (28), the stationary point is obtained regarding $\alpha_{i}$ such that

(29) $$\frac{1}{2\eta_{i}}-\frac{1}{2}C_{ii}-\frac{1}{2}m_{i}^{2}=0,$$

in which $m_{i}$ is the *i*th element of the matrix m.

From (29), we obtain the following result:

(30) $$\eta_{i}=\frac{1}{m_{i}^{2}+C_{ii}}.$$

The derivative of $\ln\left|C\right|$ is obtained regarding λ by applying Lemma 2 and (21) as follows:

(31) $$\frac{\partial }{\partial \lambda }\ln\left|C\right|=Tr(C^{-1}\frac{\partial C}{\partial \lambda })=Tr((\Lambda +\lambda \Psi^{T}\Psi )\frac{\partial }{\partial \lambda }({\left(\Lambda +\lambda \Psi^{T}\Psi \right)}^{-1})).$$

The application of Lemma 2 yields the following result.

(32) $$\frac{\partial }{\partial \lambda }\ln\left|C\right|=-Tr(\Psi^{T}\Psi C).$$

We also get

(33) $$\frac{\partial G(m)}{\partial \lambda }=\frac{1}{2}{\left\|T-\Psi m\right\|}^{2}.$$

From (32) and (33), the stationary point is obtained regarding λ by satisfying the following conditions:

(34) $$\frac{N}{\lambda }-{\left\|T-\Psi m\right\|}^{2}-Tr(\Psi^{T}\Psi C)=0.$$

From (34), we obtain

(35) $$\lambda =\frac{N}{{\left\|T-\Psi m\right\|}^{2}+Tr(\Psi^{T}\Psi C)}.$$

Let $\frac{\partial G(m)}{\partial \mu }=\Lambda \mu -\Lambda m=0$, we obtain

(36) μ=m.

From the above derivation, we know that (30), (35) and (36) are non-closed-form reestimation equations for η, λ and μ. In order to solve the problem, an iterative procedure is presented. Initially, after the mean and covariance are both computed using (20) and (21) by giving initial value η, λ and μ, respectively, we reestimate hyperparameters alternately by employing (30), (35) and (36). We again alternately employ (20) and (21) to recompute the mean and covariance, repeating this process until a suitable standard for convergence is met. Nevertheless, it remains unclear whether this approach is accurate, as outlined in the reestimate iterative procedure. To gain a clearer understanding of the iterative reestimation equations, we will turn to the EM algorithm to analyze them from both mathematical and statistical perspectives.

## 4. HPO EM Algorithm via Bayesian Optimization and Relative Entropy

The expectation maximization (EM) algorithm is a highly effective and sophisticated approach for identifying maximum likelihood solutions within probabilistic models that incorporate latent variables [35,36,37].

The EM algorithm was derived by first treating the weights as latent variables, a process that is inherently straightforward. The log marginal likelihood function is then obtained by the weight parameters marginalization over the joint distribution p(T,ω|X,η,λ,μ)

(37) L=lnp(T|X,η,λ,μ)=ln∫p(T,ω|X,η,λ,μ)dω,

where p(T,ω|X,η,λ,μ) is equal to the product of the prior ω and the likelihood given by the following result:

(38) p(T,ω|X,η,λ,μ)=p(ω|η,μ)p(T|X,ω,λ).

From Jensen’s inequality, we obtain a lower bound on L as follows:

$$\begin{array}{cc} L & =\ln\int \frac{p(T,\omega |X,\eta ,\lambda ,\mu )}{v(\omega )}v(\omega )d\omega \\ & \geq \int \ln\frac{p(T,\omega |X,\eta ,\lambda ,\mu )}{v(\omega )}v(\omega )d\omega \\ & =F(v,\eta ,\lambda ,\mu ,\theta ), \end{array}$$

where v(ω) is the variational probability distribution over the weights and θ denotes all other parameters. The EM algorithm is designed to maximize the log marginal likelihood function L by using iterative maximization of the lower bound. For the E step, we maximize F regarding the probability distribution v(ω) for fixed hyperparameters η, λ and μ. For the M step, we maximize F regarding the hyperparameters η, λ and μ for the fixed probability distribution v(ω). We rewrite the lower bound F to enhance the understanding of the E step:

(39) F=KL(v(ω)||p(ω|T,X,η,λ,μ))+L,

where KL(v(ω)||p(ω|T,X,η,λ,μ)) is the relative entropy, also called Kullback–Leibler (KL) divergence. The KL divergence is always greater than or equal to zero, which is zero only and only if the two distributions are equal. The E step corresponds thus to equating the posterior on ω with the distribution v(ω), which means v(ω)=p(ω|T,X,η,λ,μ) and F=L [38]. Given that the posterior probability distribution is also Gaussian, the E step is reduced to the computation of its mean matrix m and covariance matrix C defined by (20) and (21), respectively.

To enforce the M step, F is rewritten as a distinct form:

(40) F=∫lnp(T,ω|X,η,λ,μ)v(ω)dω−H(v(ω)),

in which H(v(ω)) clearly is irrelevant to η, λ, and μ and is the entropy of v(ω).

In order to perform the M step, from (40), we obtain

(41) $$\begin{array}{cc} F & =\int \ln p(T,\omega |X,\eta ,\lambda ,\mu )v(\omega )d\omega -H(v(\omega ))\\ & =\int \left(\ln p(\omega |\eta ,\mu \right)+\ln p(T|X,\omega ,\lambda ))v(\omega )d\omega -H(v(\omega ))\\ & =\int \ln p(\omega |\eta ,\mu )v(\omega )d\omega +\int \ln p(T|X,\omega ,\lambda )v(\omega )d\omega -H(v(\omega )) \end{array}$$

Finally, we obtain the following result:

(42) $$\begin{array}{cc} F & =\frac{1}{2}\ln\left|\Lambda \right|-\frac{M+N}{2}\ln(2\pi )-\frac{1}{2}m^{T}\Lambda m-\frac{1}{2}Tr(\Lambda C)+\mu^{T}m-\frac{1}{2}\mu^{T}\mu \\ & +\frac{N}{2}\ln\lambda -\frac{\lambda }{2}({\left\|T-\Psi m\right\|}^{2}+Tr(\Psi^{T}\Psi C))-H(v(\omega )) \end{array}$$

The proof of (42) is derived in detail in Appendix C.

The stationary point of (42) is obtained with ease regarding $\eta_{i}$, λ, and μ such that

(43) $$\frac{\partial F}{\partial \eta_{i}}=\frac{1}{2\eta_{i}}-\frac{1}{2}(m_{i}^{2}+C_{ii})=0,$$

(44) $$\frac{\partial F}{\partial \lambda }=\frac{N}{2\lambda }-\frac{1}{2}({\left\|T-\Psi m\right\|}^{2}+Tr(\Psi^{T}\Psi C))=0,$$

(45) $$\frac{\partial F}{\partial \mu }=m-\mu =0.$$

So, the update laws are obtained as follows

(46) $$\eta_{i}^{new}=\frac{1}{m_{i}^{2}+C_{ii}},$$

(47) $$\lambda^{new}=\frac{N}{{\left\|T-\Psi m\right\|}^{2}+Tr(\Psi^{T}\Psi C)},$$

(48) $$\mu^{new}=m$$

The goal of the update equations is to achieve the maximization of the log marginal likelihood by the proposed EM algorithm. This result is also the same as maximizing the evidence function.

Finally, let us provide an intuitive explanation of our proposed EM algorithm for hyperparameter optimization. Firstly, we generate initial random hyperparameters values η, λ, μ, which allows us to “guess” the initial hyperparameter. Then, we compute(guess) η, λ, μ again by (46), (47), and (48). Subsequently, the log marginal likelihood is evaluated. By repeating this process, the algorithm alternates between refining the guesses and the log marginal likelihood gradually increases until it no longer changes—that is, once it has converged, the algorithm stops making further guesses for hyperparameters η, λ, μ. This also implies that the hyperparameters have converged. Checking the convergence of the log likelihood function is easier to implement programmatically than checking the convergence of the hyperparameters. Different initial values for hyperparameters may lead to different local optima while the likelihood converges.

## 5. Experimental Set-Up

### 5.1. Synthetic Data

The parameter w either is integrated out or is considered latent variables, so after sampling from a Gaussian distribution, its goal is to construct Ψ.

We firstly generate hyperparameters η, λ, μ and $X=\left\{x_{1},\ldots ,x_{N}\right\}$ randomly and choose a suitable linear basis function $\psi_{j}(x_{n})$ to get Ψ and T. Subsequently, the EM algorithm is applied in order to maximize the likelihood or the log likelihood. The procedure is presented in detail as Algorithm 1:
**Algorithm 1: HPO EM algorithm for synthetic data**1. Generate randomly hyperparameters value η, λ, μ and the dataset $X=\left\{x_{1},\ldots ,x_{N}\right\}$
2. Choose a suitable linear basis function $\psi (x_{n})$ to get an N×M matrix Ψ by using (12).3. Generate parameters value ω randomly sampled by (2).4. Generate N-dimensional vector ε sampled randomly by the Gaussian distribution $N(0,\lambda^{-1})$ and then generate $T=(t_{1},\ldots ,t_{N})$ by (4) and (5), respectively.5. E step. Compute the mean m and covariance C using the current hyperparameter values.$m={\left(\Lambda +\lambda \Psi^{T}\Psi \right)}^{-1}(\Lambda \mu +\lambda \Psi^{T}T)$,(49)$C={\left(\Lambda +\lambda \Psi^{T}\Psi \right)}^{-1}$.(50)
6. M step. Estimate again the hyperparameters by employing the mean m and covariance C obtained by step 5 and the following update equations$\eta_{i}^{new}=\frac{1}{m_{i}^{2}+C_{ii}}$,(51)$\lambda^{new}=\frac{N}{{\left\|T-\Psi m\right\|}^{2}+Tr(\Psi^{T}\Psi C)}$,(52)$\mu^{new}=m$.(53)
7. Compute the likelihood function or the log likelihood function given by the following result: $p(T|X,\eta ,\lambda ,\mu )=\sqrt{{\left(\frac{\lambda }{2\pi }\right)}^{N}}{\left|\Lambda \right|}^{\frac{1}{2}}{\left|C\right|}^{\frac{1}{2}}\exp(-G(m))$or$\ln p(T|X,\eta ,\lambda ,\mu )=\frac{N}{2}\ln\lambda -G(m)+\frac{1}{2}\sum_{i=1}^{M}\ln\eta_{i}+\frac{1}{2}\ln\left|C\right|-\frac{N}{2}\ln(2\pi )$and then determine the convergence of the hyperparameters or the likelihood. If convergence is not satisfied, back to step 5. If the likelihood or the log likelihood converges, then the algorithm’s computational complexity is O(M).

We apply Algorithm 1 to optimize hyperparameters value η, λ, and μ, where the linear basis function ψ(x) is a Gaussian basis function. We have difficulty judging the convergence of hyperparameter values η, λ, and μ, so we turn to judge the convergence of the likelihood or the log likelihood. It is reasonable because the convergence of hyperparameter values η, λ, and μ means the likelihood or the log likelihood keeps constant; in other words, the convergence of the likelihood or the log likelihood. This is much easier to implement programmatically.

**Figure 1:**
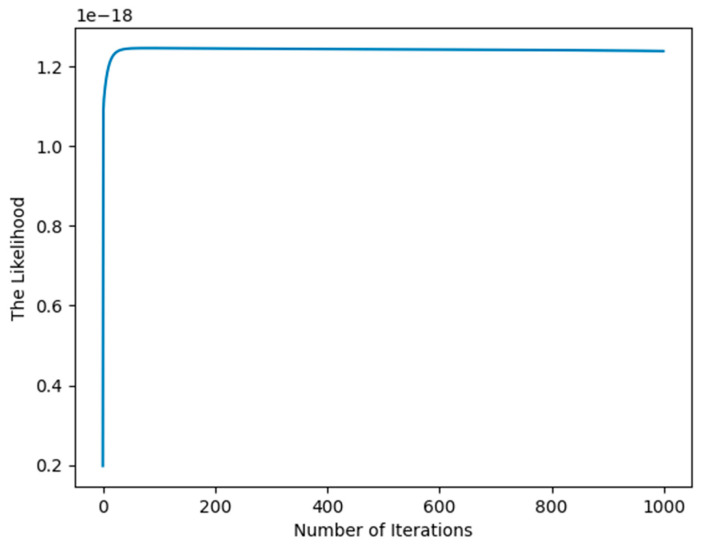
Illustration of the convergence of the EM algorithm for synthetic data.

From Figure 1, we see that after approximately 10 iterations, the likelihood converges, so we can draw the conclusion that the EM algorithm has the advantage of fast convergence, which comes from strict interpretation mathematically and statistically.

**Figure 2:**
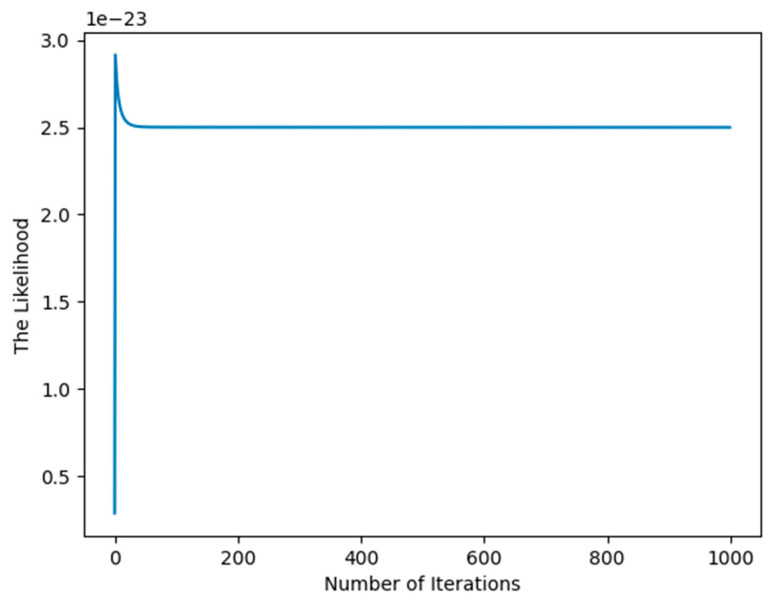
Illustration of singular covariance C affecting the increase in the likelihood.

In the process of experimental set-up, the covariance C of the posterior distribution defined by (50) may easily be a singular matrix, so we have to use a pseudo-inverse matrix to evaluate C, which may produce the imprecise result that affects the increase in the likelihood function as shown in Figure 2.

The singularity for C arises from the initial value of the hyperparameter values η, λ, and μ, the simplest and intuitive method is to randomly generate initial values of η, λ, and μ again and again when the singularity occurs until the convergence criterion is met like Figure 2.

**Figure 3:**
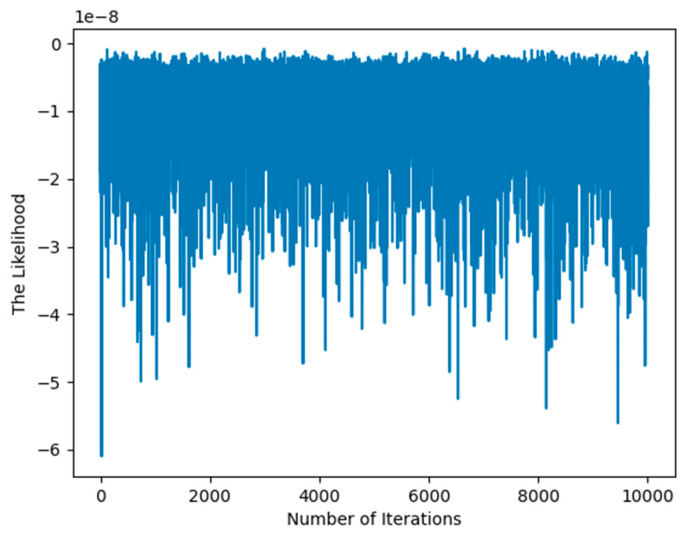
Illustration of convergence of the likelihood base on PSO.

Particle swarm optimization (PSO) is an intuitive and computationally efficient metaheuristic that is highly effective for hyperparameter optimization across a wide range of machine learning models. For the same likelihood function, we use PSO to optimize the hyperparameter values η, λ, and μ, as shown in Figure 3. After 10,000 iterations, the likelihood function still has not converged. This highlights the advantage of our proposed method’s fast convergence, which is attributed to its rigorous mathematical foundation.

### 5.2. The Diabetes Dataset

The diabetes dataset contains data from 442 diabetes patients, with each patient having measurements for 10 baseline variables, including age, sex, body mass index, average blood pressure, and six blood serum measurements. In addition, each patient has a response variable that represents a quantitative measure of disease progression one year after the baseline. This dataset is commonly used for predicting the progression of the disease and is one of the most frequently used datasets in machine learning. It is particularly well suited for research on regression problems.

Firstly, from the diabetes dataset, we obtain the input dataset $X=\left\{x_{1},\ldots ,x_{442}\right\}$, a 8×442 matrix and the target value $T=(t_{1},\ldots ,t_{442})$. We then generate hyperparameters η, λ, μ randomly and choose a suitable linear basis function $\psi_{j}(x_{n})$ to get Ψ. Subsequently, the EM algorithm is applied in order to maximize the likelihood or the log likelihood. The procedure is presented in detail as Algorithm 2:
**Algorithm 2: HPO EM algorithm for the diabetes dataset**1. Generate randomly hyperparameters value η, λ, μ.2. Choose a suitable linear basis function $\psi (x_{n})$ to get an 8×442 matrix Ψ by using (12).3. E step. Compute the mean m and covariance C using the current hyperparameter values.$m={\left(\Lambda +\lambda \Psi^{T}\Psi \right)}^{-1}(\Lambda \mu +\lambda \Psi^{T}T),$(54)$C={\left(\Lambda +\lambda \Psi^{T}\Psi \right)}^{-1}.$(55)
4. M step. Estimate again the hyperparameters by employing the mean m and covariance C obtained by step 3 and the following update equations$\eta_{i}^{new}=\frac{1}{m_{i}^{2}+C_{ii}},$(56)$\lambda^{new}=\frac{N}{{\left\|T-\Psi m\right\|}^{2}+Tr(\Psi^{T}\Psi C)},$(57)$\mu^{new}=m.$(58)
5. Compute the likelihood function or log likelihood function given by the following result:$p(T|X,\eta ,\lambda ,\mu )=\sqrt{{\left(\frac{\lambda }{2\pi }\right)}^{N}}{\left|\Lambda \right|}^{\frac{1}{2}}{\left|C\right|}^{\frac{1}{2}}\exp(-G(m))$or$\ln p(T|X,\eta ,\lambda ,\mu )=\frac{N}{2}\ln\lambda -G(m)+\frac{1}{2}\sum_{i=1}^{M}\ln\eta_{i}+\frac{1}{2}\ln\left|C\right|-\frac{N}{2}\ln(2\pi )$and then determine the convergence of the hyperparameters or the likelihood. If convergence criterion is not satisfied, go back to step 3.

**Figure 4:**
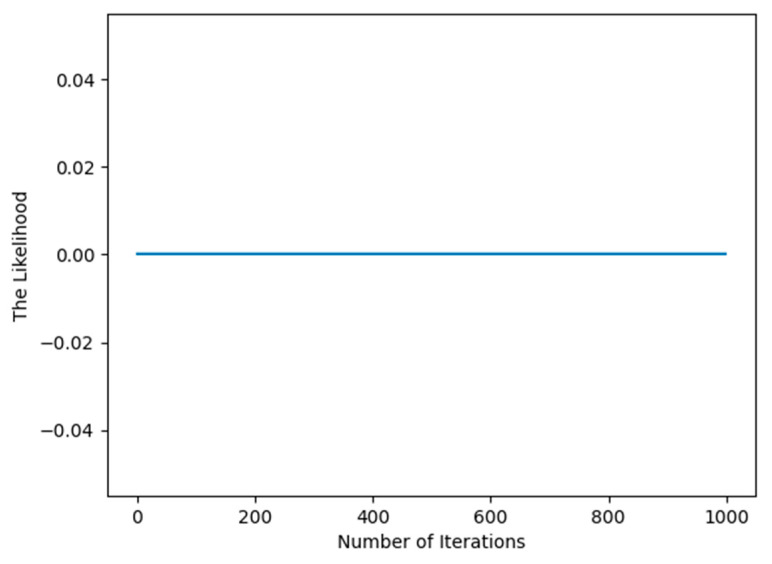
Illustration of the convergence of the EM algorithm for the diabetes dataset.

From Figure 4, we can also draw the same conclusion that the EM algorithm has the advantage of fast convergence for the famous dataset of machine learning after the first iteration.

## 6. Conclusions

In this paper, we present the general Gaussian weight prior rather than zero-mean Gaussian weight priors for the hyperparameter optimization in the field of machine learning. We firstly derive non-closed-form iterative reestimation equations for hyperparameters by evidence function maximization. Though we know how to optimize the hyperparameters, we can’t understand the iterative reestimation equations clearly. To better understand non-closed-form reestimation equations more clearly, we have to resort to the EM algorithm. After using Bayesian theory and optimization, the EM algorithm partitions the iterative reestimation equations into E and M steps, which provides a clear interpretation for the iterative reestimation equations both mathematically and statistically. The experimental result shows the effectiveness of the EM algorithm of hyperparameter optimization, and the EM algorithm also has the advantage of fast convergence, except that the variance C of the posterior distribution defined by (50) is a singular matrix, which affects the increase in the likelihood.

## Data Availability

The datasets used and/or analyzed during the current study available from the corresponding author on reasonable request.

## Appendix A

From (17) and (18), the following result is obtained

$$p(T|X,\omega ,\lambda )=N(T|\Psi \omega ,\lambda^{-1})$$

and

$$p(\omega |\eta ,\mu )=N(\omega |\mu ,\Lambda^{-1})$$

From (10), (11), and using $m_{0}=\mu$ and $C_{0}=\Lambda^{-1}$, we obtain

$$C^{-1}=C_{N}^{-1}=C_{0}^{-1}+\lambda \Psi^{T}\Psi =\Lambda +\lambda \Psi^{T}\Psi ,$$

$$m=C_{N}(C_{0}^{-1}m_{0}+\lambda \Psi^{T}T)={\left(\Lambda +\lambda \Psi^{T}\Psi \right)}^{-1}(\Lambda \mu +\lambda \Psi^{T}T)$$

## Appendix B

By substituting (17) and (18) into (22), the following result is obtained

$$p(T|X,\eta ,\lambda ,\mu )=\sqrt{{\left(\frac{\lambda }{2\pi }\right)}^{N}}\frac{{\left|\Lambda \right|}^{\frac{1}{2}}}{\sqrt{{\left(2\pi \right)}^{M}}}\int \exp(-G(\omega ))d\omega ,$$

together with

$$\begin{array}{cc} G(\omega ) & =\frac{\lambda }{2}{\left\|T-\Psi \omega \right\|}^{2}+\frac{1}{2}{\left(\omega -\mu \right)}^{T}\Lambda (\omega -\mu )\\ & =\frac{\lambda }{2}{(T-\Psi \omega )}^{T}(T-\Psi \omega )+\frac{1}{2}\omega^{T}\Lambda \omega -\omega^{T}\Lambda \mu +\frac{1}{2}\mu^{T}\Lambda \mu \\ & =\frac{1}{2}\omega^{T}(\Lambda +\lambda \Psi^{T}\Psi )\omega -\omega^{T}(\lambda \Psi^{T}T+\Lambda \mu )+\frac{1}{2}\mu^{T}\Lambda \mu +\frac{\lambda }{2}T^{T}T. \end{array}$$

Firstly, we compute the gradient of ∇G(ω) and let it be zero. Then, we obtain the stationary point of G(ω) as follows:

$$\nabla G(\omega )=(\Lambda +\lambda \Psi^{T}\Psi )\omega -(\Lambda \mu +\lambda \Psi^{T}T),$$

$$\nabla \nabla G(\omega )=\Lambda +\lambda \Psi^{T}\Psi =C^{-1}.$$

Let

$$\nabla G(\omega )=(\Lambda +\lambda \Psi^{T}\Psi )\omega -(\Lambda +\lambda \Psi^{T}T)=0,$$

Then, we obtain the following result:

$$\omega ={\left(\Lambda +\lambda \Psi^{T}\Psi \right)}^{-1}(\Lambda \mu +\lambda \Psi^{T}T)=m.$$

We obtain the following result by using Corollary 1:

$$G(\omega )=G(m)+\frac{1}{2}{\left(\omega -m\right)}^{T}C^{-1}(\omega -m)$$

along with

$$G(m)=\frac{\lambda }{2}{\left\|T-\Psi m\right\|}^{2}+\frac{1}{2}{\left(m-\mu \right)}^{T}\Lambda (m-\mu ).$$

Thus, we obtain

$$\begin{array}{cc} p(T|X,\eta ,\lambda ,\mu ) & =\sqrt{{\left(\frac{\lambda }{2\pi }\right)}^{N}}\frac{\sqrt{\left|\Lambda \right|}}{\sqrt{{\left(2\pi \right)}^{M}}}\exp(-G(m))\left(\int \exp(-\frac{1}{2}{\left(\omega -m\right)}^{T}C^{-1}(\omega -m))d\omega \right)\\ & =\sqrt{{\left(\frac{\lambda }{2\pi }\right)}^{N}}{\left|\Lambda \right|}^{\frac{1}{2}}{\left|C\right|}^{\frac{1}{2}}\exp(-G(m)), \end{array}$$

where we have employed the following result:

$$\frac{1}{\sqrt{{\left(2\pi \right)}^{M}}{\left|C\right|}^{\frac{1}{2}}}\int \exp(-\frac{1}{2}{\left(\omega -m\right)}^{T}C^{-1}(\omega -m))d\omega =1.$$

## Appendix C

We obtain the following the result from the first term of (41)

$$\begin{array}{c} \int \ln p(\omega |\eta ,\mu )v(\omega )d\omega \\ \;=\int \left(\frac{1}{2}\ln\left|\Lambda \right|-\frac{M}{2}\ln(2\pi )-\frac{1}{2}{\left(\omega -\mu \right)}^{T}\Lambda (\omega -\mu \right))v(\omega )d\omega \\ \;=\int (\frac{1}{2}\ln\left|\Lambda \right|-\frac{M}{2}\ln(2\pi ))v(\omega )d\omega -\frac{1}{2}\int {\left(\omega -\mu \right)}^{T}\Lambda (\omega -\mu )v(\omega )d\omega \\ \;=\frac{1}{2}\ln\left|\Lambda \right|-\frac{M}{2}\ln(2\pi )-\frac{1}{2}\int {\left(\omega -\mu \right)}^{T}\Lambda (\omega -\mu )v(\omega )d\omega , \end{array}$$

where ∫v(ω)dω=1 is used.

We get the following result:

$$\begin{array}{c} \int {\left(\omega -\mu \right)}^{T}\Lambda (\omega -\mu )v(\omega )d\omega \\ \;=\int Tr(\Lambda (\omega -\mu ){\left(\omega -\mu \right)}^{T})v(\omega )d\omega \\ \;=\int Tr(\Lambda \omega \omega^{T}-\omega \mu^{T}-\mu \omega^{T}+\mu \mu^{T})v(\omega )d\omega \\ \;=\int \left(Tr(\Lambda \omega \omega^{T})-2Tr(\omega \mu^{T})+Tr(\mu \mu^{T}))v(\omega \right)d\omega \\ \;=Tr(\Lambda \int \omega \omega^{T}v(\omega )d\omega )-2Tr((\int \omega v(\omega )d\omega )\mu^{T})+Tr(\int \mu \mu^{T}v(\omega )d\omega )\\ \;=Tr(\Lambda E_{v}(\omega \omega^{T}))-2Tr(E_{v}(\omega )\mu^{T})+Tr(\mu \mu^{T})\\ \;=Tr(\Lambda (mm^{T}+C))-2Tr(m\mu^{T})+Tr(\mu \mu^{T})\\ \;=Tr(\Lambda mm^{T})+Tr(\Lambda C)-2Tr(m\mu^{T})+Tr(\mu \mu^{T})\\ \;=m^{T}\Lambda m+Tr(\Lambda C)-2\mu^{T}m+\mu^{T}\mu , \end{array}$$

where we have used $E_{v}(\omega \omega^{T})=mm^{T}+C$, where $E_{v}(\cdot )$ is the expectation over the given probability distribution v(ω). Tr(⋅) is the trace of a matrix.

Finally, we get

$$\begin{array}{c} \int \ln p(\omega |\eta ,\mu )v(\omega )d\omega \\ \;=\frac{1}{2}\ln\left|\Lambda \right|-\frac{M}{2}\ln(2\pi )-\frac{1}{2}m^{T}\Lambda m-\frac{1}{2}Tr(\Lambda C)+\mu^{T}m-\frac{1}{2}\mu^{T}\mu . \end{array}$$

The second term of (41) is now computed, and then the following result is obtained:

$$\begin{array}{c} \int \ln p(T|X,\omega ,\lambda )v(\omega )d\omega \\ \;=\int \ln(\sqrt{{\left(\frac{\lambda }{2\pi }\right)}^{N}}\exp(-\frac{\lambda }{2}{\left\|T-\Psi \omega \right\|}^{2}))v(\omega )d\omega \\ \;=\int \left(\frac{N}{2}\ln\lambda -\frac{N}{2}\ln(2\pi ))v(\omega \right)d\omega -\frac{\lambda }{2}\int {\left\|T-\Psi \omega \right\|}^{2}v(\omega )d\omega \\ \;=\frac{N}{2}\ln\lambda -\frac{N}{2}\ln(2\pi )-\frac{\lambda }{2}\int {\left\|T-\Psi \omega \right\|}^{2}v(\omega )d\omega \\ \;=\frac{N}{2}\ln\lambda -\frac{N}{2}\ln(2\pi )-\frac{\lambda }{2}\int {\left(T-\Psi \omega \right)}^{T}(T-\Psi \omega )v(\omega )d\omega \\ \;=\frac{N}{2}\ln\lambda -\frac{N}{2}\ln(2\pi )-\frac{\lambda }{2}\int \left(T^{T}T-2\omega^{T}\Psi^{T}T+\omega^{T}\Psi^{T}\Psi \omega )v(\omega \right)d\omega . \end{array}$$

The following result is obtained

$$\begin{array}{c} \int \left(T^{T}T-2\omega^{T}\Psi^{T}T+\omega^{T}\Psi^{T}\Psi \omega \right)v(\omega )d\omega \\ \;=\int T^{T}Tv(\omega )d\omega -2\int \omega^{T}\Psi^{T}Tv(\omega )d\omega +\int \omega^{T}\Psi^{T}\Psi \omega v(\omega )d\omega \\ \;=T^{T}T-2\int \omega^{T}\Psi^{T}Tv(\omega )d\omega +\int Tr(\Psi \omega \omega^{T}\Psi^{T})v(\omega )d\omega \\ \;=T^{T}T-2\int \omega^{T}\Psi^{T}Tv(\omega )d\omega +Tr\int \Psi \omega \omega^{T}\Psi^{T}v(\omega )d\omega \\ \;=T^{T}T-2(\int \omega^{T}v(\omega )d\omega )\Psi^{T}T+Tr(\Psi (\int \omega \omega^{T}v(\omega )d\omega )\Psi^{T})\\ \;=T^{T}T-2E_{v}(\omega^{T})\Psi^{T}T+Tr(\Psi E_{v}(\omega \omega^{T})\Psi^{T})\\ \;=T^{T}T-2m^{T}\Psi^{T}T+Tr(\Psi (mm^{T}+C)\Psi^{T})\\ \;=T^{T}T-2m^{T}\Psi^{T}T+Tr(\Psi mm^{T}\Psi^{T}+\Psi C\Psi^{T})\\ \;=T^{T}T-2m^{T}\Psi^{T}T+Tr(\Psi mm^{T}\Psi^{T})+Tr(\Psi C\Psi^{T})\\ \;=T^{T}T-2m^{T}\Psi^{T}T+m^{T}\Psi^{T}\Psi m+Tr(\Psi C\Psi^{T})\\ \;={\left(T-\Psi m\right)}^{T}(T-\Psi m)+Tr(\Psi C\Psi^{T})\\ \;={\left\|T-\Psi m\right\|}^{2}+Tr(\Psi C\Psi^{T})\\ \;={\left\|T-\Psi m\right\|}^{2}+Tr(C\Psi^{T}\Psi )\\ \;={\left\|T-\Psi m\right\|}^{2}+Tr(\Psi^{T}\Psi C), \end{array}$$

where $E_{v}(\omega \omega^{T})=mm^{T}+C$ is used.

The result is obtained as follows:

$$\begin{array}{c} \int \ln p(T|X,\omega ,\lambda )v(\omega )d\omega \\ \;=Tr(\Psi^{T}\Psi C)+\frac{N}{2}(\ln\lambda -\ln(2\pi ))-\frac{\lambda }{2}({\left\|T-\Psi m\right\|}^{2}). \end{array}$$

Finally, we get

$$\begin{array}{cc} F & =\frac{1}{2}\ln\left|\Lambda \right|-\frac{M+N}{2}\ln(2\pi )-\frac{1}{2}m^{T}\Lambda m-\frac{1}{2}Tr(\Lambda C)+\mu^{T}m-\frac{1}{2}\mu^{T}\mu \\ & +\frac{N}{2}\ln\lambda -\frac{\lambda }{2}({\left\|T-\Psi m\right\|}^{2}+Tr(\Psi^{T}\Psi C))-H(v(\omega )) \end{array}$$
